# Enforcement of State Indoor Tanning Laws in the United States

**Published:** 2008-09-15

**Authors:** Joni A Mayer, Katherine D Hoerster, Latrice C Pichon, Debra A Rubio, Susan I Woodruff, Jean L Forster

**Affiliations:** Graduate School of Public Health, San Diego State University; Joint Doctoral Program in Clinical Psychology, San Diego State University/University of California; Joint Doctoral Program in Public Health, Health Behavior, San Diego State University/University of California; School of Social Work, San Diego State University, San Diego, California; School of Social Work, San Diego State University, San Diego, California. At the time of this research, Susan I. Woodruff was affiliated with the Graduate School of Public Health, San Diego State University, San Diego, California; School of Public Health, University of Minnesota, Minneapolis, Minnesota

## Abstract

**Introduction:**

Twenty-eight US states have passed legislation for indoor tanning facilities. To our knowledge, whether these state laws are actually enforced has not been evaluated previously in all 28 states. Therefore, we interviewed key informants in these states to assess enforcement practices.

**Methods:**

Two trained interviewers used a structured survey instrument to interview 28 key informants who were knowledgeable about enforcement practices for laws regarding indoor tanning. Respondents provided information specific to the most populous city in their states.

**Results:**

Licensure for indoor tanning businesses was required in 22 of the 28 cities. Slightly less than half of the cities gave citations to tanning facilities that violated state law. Approximately 32% of the cities did not inspect indoor tanning facilities for compliance with state law, and another 32% conducted inspections less than annually. Of those cities that inspected at all, most conducted unannounced inspections.

**Conclusion:**

The relatively low rates of annual inspections and citations are of concern. We recommend that future studies assess whether legislation, enforcement practices, or a combination of the 2 affects the practices of indoor tanning facilities or of consumers.

## Introduction

Indoor tanning with UV radiation lamps has been linked to melanoma (1), squamous cell carcinoma (1), molecular damage associated with skin cancer (2), and other acute damage to eyes and skin (3,4). Commercial indoor tanning facilities are prevalent in the United States (5), and "all-you-can-tan" discount pricing packages make indoor tanning inexpensive (6). The rates of indoor tanning for teen girls in the United States are high (7-10); in a national sample, approximately 40% of 17- to 18-year-old girls had used indoor tanning in the past year (7).

Some US states have passed legislation regulating indoor tanning facilities, with the intent of reducing risks to consumers. Ongoing systematic updates on the number and content of these laws have been provided, focusing on youth access restrictions (11-13). A recent report quantified the stringency of state indoor tanning legislation in the 28 states that had a state law as of 2006 (14). However, in order to assess the level at which the laws are implemented and the effect of these laws on the industry and consumers, information about enforcement practices is needed. Consequently, we conducted telephone interviews of key informants in states with indoor tanning legislation to assess enforcement practices.

## Methods

### Settings and participants

The CITY100 (Correlates of Indoor Tanning in Youth) project assesses factors that may influence use of indoor tanning by adolescents (10,14); 1 objective is to better understand current legislation that pertains to indoor tanning. In the current study, we targeted the most populous city in each of the 28 states to evaluate how state laws are enforced at the local level.

Our goal was to interview, by telephone, the person who was the most knowledgeable about enforcement practices in each city or county. From a list of contacts for each state's legislation presented on a Web site operated by the tanning industry (15), we telephoned these contacts (typically at the state or county health department) and asked them to identify the best key informant. The process for identifying each city's respondent continued until we found a knowledgeable potential respondent. We then mailed an introductory letter to each potential respondent that explained the purpose of the study and its voluntary nature and assured anonymity of the respondent and that data would not be linked to the city's name in any published reports. Approximately 1 week after mailing the letter, we attempted to contact informants until we reached them and they completed the interview. The 2 interviewers (K.D.H. and L.C.P.) had previous experience in conducting telephone interviews and received training for this study.

### Survey

The survey questions were based on a combination of previous study in this area (16), expert opinion about measuring enforcement activities in the tobacco control area (a good model for indoor tanning), and select enforcement and monitoring activities mentioned in the indoor tanning laws (14). Initially, we developed a longer version of the survey that asked for specific data on various activities (eg, number of facilities inspected in the previous year, number of complaints received). That version assumed a high level of inspection and other enforcement activities, assumed that enforcement agencies kept detailed records of those activities, and requested that informants obtain that information before the interview and provide it during the interview. During the informant identification process, we became aware that the level of enforcement activities was fairly low. Therefore, to better match the depth of our assessment to actual practices, reduce the amount of work required of respondents, and achieve a higher response rate, we retained only the basic items and eliminated the more elaborate, labor-intensive items.

The following factors were assessed: number of staff allocated to carry out enforcement activities in the city/county; whether indoor tanning businesses were required to have a license; frequency of inspections (in absence of a complaint); whether inspections were announced in advance; whether inspection included review of customer records and, if so, whether customer's age, parental consent forms, number and dates of tanning sessions, and duration of sessions were examined; and whether businesses received citations when they violated the law. We also assessed the types of penalties for selling sessions to underage youth or not obtaining parental permission for minors and whether graduated penalties (more severe penalties for each successive violation) were used. A copy of the survey is provided in the Appendix.

A draft of the survey was reviewed for clarity by 2 public health department professionals. All survey procedures and materials were approved by the institutional review board at San Diego State University. Interviews were conducted in April and May of 2007.

### Statistical analysis

Descriptive statistics, including frequencies or means, were computed for each variable. Additionally, we conducted bivariate tests (χ^2^ and correlations) to assess the associations between the stringency of the written law (14) and reported enforcement practices. Specifically, we examined the relationship between reported inspection frequency and overall law stringency score, youth access subscore, enforcement subscore, and 1 individual inspection item. For the items about penalties specific to youth access, we computed frequencies for only the 21 cities in states with youth access laws. We did not perform multivariate tests because of the small sample size. All analyses were conducted in SPSS 13.0 (SPSS Inc, Chicago, Illinois).

## Results

### Response rates and respondent characteristics

We identified 28 respondents (1 for the most populous city in each of the 28 targeted states) who confirmed that they were knowledgeable about enforcement practices. Data were obtained for all 28 cities. If respondents told the interviewer that their states had no law (n = 5) or that the cities engaged in no enforcement activities (n = 2), the interviewer contacted additional informants to confirm that laws were not enforced. The interviewer then coded the remaining survey items to indicate nonperformance of enforcement activities.

### Enforcement resources and practices

More than three-fourths of the respondents were employed by a state or local health agency (Table 1). The organizations that employed the respondents also constituted the primary enforcement entity for the state indoor tanning legislation in the designated city.

The number of full-time employees available for inspections and other enforcement activities ranged from 0 to 15, with a mean of 3.29 (standard deviation 3.89) staff and a median of 2. Approximately 29% of the cities had no full-time enforcement staff (Table 2). Licensure for indoor tanning businesses was required in most cities. Slightly less than half of the cities gave citations (ie, penalties) to tanning facilities that violated the state law. Approximately 32% of the cities did not inspect indoor tanning facilities for compliance with the state law, and another 32% conducted inspections less than annually. Of those cities that inspected, most conducted unannounced inspections.

Of the 19 cities that conducted inspections, most reviewed customer records as part of the inspection process. Of these, most reviewed information about customers' ages, parental consent forms, number and dates of tanning sessions, and tanning session duration (Table 3).

Of the 21 cities in states that had youth access laws, approximately half penalized these violations (Table 4). Warnings, monetary fines, and license suspensions were used for both kinds of youth access violations, with no strong predominance by type of penalty. Of the cities that penalized violations, most gave graduated penalties for each of the youth access–related violations, in which each additional violation results in a larger penalty.

### Bivariate associations

We conducted Pearson correlational tests between inspection frequency and the variables from an earlier assessment of state indoor tanning laws (14). These correlations (N = 28) were 0.51 (*P* = .006) for enforcement subscore, 0.34 (*P* = .075) for minor's access stringency subscore, and 0.58 (*P* = .001) for overall law stringency score. Reported inspection frequency was positively correlated with the number of full-time enforcement staff reported by the respondent (*r* = 0.48, *P* = .011). We then dichotomized reported inspection frequency (less than annually vs at least annually) and the individual inspection item score from the earlier analysis of state laws (less strict vs more strict). These variables were significantly associated (χ^2^ = 5.18, *P* = .023). Of cities whose laws on inspections were less strict (n = 21), only 23.8% conducted inspections at least annually. Of those whose inspection requirement was stricter (n = 7), 71.4% conducted inspections at least annually. A license requirement in the written law was significantly associated with actual (self-reported) license requirement (χ^2^ = 5.06, *P* = .024). In cities in which the state law did not mention licensure (n = 6), 50% required licensure, whereas in cities whose law mentioned licensure (n = 21), 90.5% required licensure.

## Discussion

To our knowledge, this article is 1 of only 3 to report actual enforcement practices related to state indoor tanning laws (16,17) and the only article to date that provides enforcement information for all 28 states. Our data indicate that routine annual inspections, which are a prerequisite for other enforcement activities such as levying penalties for violations, are not conducted in 64% of the cities. However, for those cities that conduct regular inspections, most conduct unannounced inspections, which likely increases their effectiveness. Additionally, the annual inspections routinely included review of client records and encompassed information that may reflect UV radiation exposure levels (eg, duration and frequency of sessions) and youth access (eg, customer age and parental consent forms). Thus, the annual inspections appear to be of high quality. The relationship between inspection frequency and staffing level suggests that cities that do not conduct annual inspections need more resources. However, we cannot infer causality because of the study's cross-sectional design. Results from a study of sanitarians within 1 large metropolitan area in both Massachusetts (21 municipalities of Boston) and Minnesota (21 jurisdictions of the Twin Cities) indicated the rates of routine inspections by Massachusetts agencies were higher than those in Minnesota (89.9% vs 28.6%) (16). Responses from state health department staff in Texas, Illinois, and Wisconsin showed a large amount of variability (for each state as a whole) on both penalties to facilities for youth access violations and facility inspection/auditing practices (17).

The low rates of penalizing any violation and youth access violations are also cause for concern. As is the case with enforcement activities regarding tobacco and alcohol control, businesses are less likely to comply with age-of-sale laws if noncompliance is not penalized (18,19).

The strong association between various aspects of the law and enforcement activities is encouraging. However, in the 7 states with more stringent inspection requirements, the inspection requirements tended to overestimate the actual level of inspections. Moreover, 5 respondents incorrectly reported that their states, at the time of the interview, had no law on indoor tanning. Therefore, even though some states had laws, they were not being enforced even minimally.

One limitation of this study is that the data were based on the report of enforcement professionals, and we did not attempt to verify them with other measures such as interviews with tanning facility managers. Although we assured their anonymity, respondents may have overreported enforcement practices. Second, we used the most populous city in each state to represent enforcement of the state law, so our findings may not generalize to other cities and rural areas in each state. Initially, in 18 of the 28 states, we interviewed participants about the enforcement practices of at least 1 additional large city in that state. However, because the key enforcement practices were almost perfectly consistent between cities in each state, we ultimately used the "largest city in state" approach. That experience leads us to believe that the data for each city generalize to other large cities in each state. Finally, we neglected to ask about each state's licensing fees, if any; such fees could help fund enforcement activities (20).

Strengths of our study included an optimal response rate and our access to reliable data on the stringency of each state's law (14). These data facilitated comparisons between "ideal" and "real" enforcement activities. As noted earlier, we promised respondents that we would not link published data to individuals or cities; this assurance of anonymity probably improved both our response rate and the accuracy of the data. Even though we are unable to reveal which states were enforcing at lower levels, we will be providing each respondent with feedback on how the city's enforcement level compares with enforcement for all other cities combined. For states in which enforcement is low, the feedback may increase enforcement practices.

In tobacco control, antitobacco organizations historically focused their efforts on passing new laws, but enforcement of existing laws is viewed by many to have been critical to reducing tobacco use (21-26). In the field of indoor tanning legislation, we cannot say whether legislation, enforcement practices, or a combination of the 2 has any effect on the practices of indoor tanning facilities or of consumers. Therefore, we recommend that future evaluations of indoor tanning legislation measure not only the written law but also its implementation and enforcement; research should also attempt to assess the relationship between legislation strictness/enforcement level and the practices of businesses and consumers, especially adolescent consumers.

## Figures and Tables

**Table 1:** Respondent and Organization Characteristics (N = 28), CITY100 Enforcement Survey, April and May 2007

**Variable**	n (%)
**Sex**	
Women	7 (25.0)
Men	21 (75.0)
**Organization**	
State health, environmental health, or radiologic health agency	15 (53.6)
City or county health, environmental health, or radiologic health agency	7 (25.0)
State cosmetology board	4 (14.3)
Other state agency	2 (7.1)
**Occupation/title**	
Department head or director	6 (21.4)
Supervisor or manager	6 (21.4)
Health physicist	4 (14.3)
Environmental health or industrial hygiene	3 (10.7)
Sanitarian	3 (10.7)
Inspector	2 (7.1)
Other^a^	4 (14.3)

Abbreviation: CITY100, Correlates of Indoor Tanning in Youth.

a These titles included investigator, board administrator, regulatory enforcement, and compliance officer.

**Table 2:** Indoor Tanning Legislation Enforcement Resources and General Practices (N = 28), CITY100 Enforcement Survey, April and May 2007

**Variable**	n (%)
**No. of full-time enforcement staff**	
0	8 (28.6)
1-2	7 (25.0)
3-4	7 (25.0)
≥5	6 (21.4)
**Licensure required**	
No	5 (17.9)
Yes	22 (78.6)
Don't know	1 (3.6)
**Give citation if facility violates law**	
No	14 (50.0)
Yes	13 (46.4)
Don't know	1 (3.6)
**Inspection schedule**	
Never	9 (32.1)
Less than annually	9 (32.1)
Annually	6 (21.4)
Twice a year	4 (14.3)
**Announce inspection in advance^a^**	
Never/rarely	17 (89.5)
Sometimes	2 (10.5)
Often/always	0

Abbreviation: CITY100, Correlates of Indoor Tanning in Youth.

a Based on the 19 cities that ever conducted inspections.

**Table 3:** Indoor Tanning Facility Inspection Practices Related to Customer Records, CITY100 Enforcement Survey, April and May 2007

Practice	Frequency		

	Never/Rarely n (%)	Sometimes n (%)	Often/Always n (%)
Inspection includes customer record review^a^	2 (10.5)	2 (10.5)	15 (78.9)
Review includes customer's age^b^	1 (5.9)	0	16 (94.1)
Review includes parental consent forms^b^	2 (11.8)	0	15 (88.2)
Review includes number and dates of sessions^b^	1 (5.9)	3 (17.6)	13 (76.5)
Review includes session duration^b^	2 (11.8)	1 (5.9)	14 (82.4)

Abbreviation: CITY100, Correlates of Indoor Tanning in Youth.

a Based on the 19 cities that ever conducted inspections.

b Based on the 17 cities that included customer record review when inspecting.

**Table 4:** Penalties for Youth Access Violations in States With Indoor Tanning Access Laws, CITY100 Enforcement Survey, April and May, 2007

Penalty	Violation	

	Selling Sessions to Underage Youth n (%)^a^	Not Obtaining Parental Consent for Minors n (%)^b^
**Penalty type**		
Any type	11 (55.0)	10 (47.6)
Warning	10 (50.0)	9 (42.9)
Monetary fine	8 (40.0)	8 (38.1)
License suspension	7 (35.0)	7 (33.3)
Other	3 (15.0)	1 (4.8)
**Graduated penalties given^c^**		
No	4 (36.4)	3 (30.0)
Yes	7 (63.6)	7 (70.0)

Abbreviation: CITY100, Correlates of Indoor Tanning in Youth.

a Based on 20 respondents because of missing data.

b Based on 21 respondents.

c Based on those respondents whose cities gave any type of penalty for the violation.

## Appendix: CITY100 Enforcement Survey

### Study Introduction

This interview is part of the CITY100 Indoor Tanning Project. CITY100 (Correlates of Indoor Tanning in Youth) is a project funded by the National Cancer Institute that will help us better understand the factors that influence teens to use indoor tanning. The project is based at the Graduate School of Public Health at San Diego State University and is focusing on over 100 cities in the US. One goal of CITY100 is to evaluate enforcement activities in cities located in states with indoor tanning laws. Your state has a law governing indoor tanning.

If you decide to participate, I will ask you questions about the enforcement activities in [city/county], such as inspections of indoor tanning facilities. **These are activities at the city or county level to enforce the state law.** We would like you to participate, irrespective of whether your city has many or few enforcement activities. The interview will take only around 5 to 7 minutes. The researcher in charge of this study is Dr Joni Mayer; you may have her phone number if you wish to write it down. Collect calls are accepted. She will be able to answer any questions you have. Or if you have any questions now, I can answer them for you. I want to assure you that your responses in this interview will not be linked with your name, city, or county in any written reports or publications. All data will be kept in locked file cabinets, and only the CITY100 research staff will have access to the data. Participation is voluntary, and you are free to end the interview at any point.

### Resources (R)

I'd like to first ask you about your employment, and [city's/county's] resources related to regulating indoor tanning businesses.

R1a. What is the agency you work for?

R1b. What department within that agency?

R1c. Is your job at the

- City level?
- County level?
- State level?
- None of these? Describe.

R2. What is your occupation and/or job title?

R3. How many agencies are responsible for enforcing the state's indoor tanning law in [city/county]?

- None/no enforcement
- One
- Two or more
- Don't know
- Refused
- No state law

R4. The **primary** enforcement agency—is it at the

- City-level? Name:
- County-level? Name:
- State-level? Name:
- None of these? Name:

R5. How many full-time staff (or FTE) are allocated to carry out the inspections and/or other enforcement activities in [city/county]?

- Gave a number: ___
- Don't know
- Refused
- Not applicable

### Licensure (L)

L1. Are indoor tanning businesses in [city] required to have a license?

- No
- Yes
- Don't know
- Refused
- Not applicable

### Inspections (I)

Now I'd like to ask you about inspection procedures.

I1. In the absence of a complaint, how often is a tanning facility inspected in [city/county]?

- Never **(go to question P1)**
- Less than once a year
- Once a year
- Twice a year
- More than twice a year
- Other (describe)
- Don't know
- Refused
- Not applicable

I2. How often is the inspection announced in advance to the tanning facility that will be inspected?

- Never/rarely
- Sometimes
- Often/always
- Don't know
- Refused
- Not applicable

I3. Please tell me how often an inspection includes the review of customer records.

- Never/rarely
- Sometimes
- Often/always
- Don't know
- Refused
- Not applicable

I4. **(If the answer to I3 is sometimes, often, or always)** When customer records are reviewed, how often are the following looked at?

I4a. Age of customers

- Never/rarely
- Sometimes
- Often/always
- Don't know
- Refused
- Not applicable

I4b. Parental consent forms

- Never/rarely
- Sometimes
- Often/always
- Don't know
- Refused
- Not applicable

I4c. Number and dates of tanning sessions

- Never/rarely
- Sometimes
- Often/always
- Don't know
- Refused
- Not applicable

I4d. Duration of tanning sessions

- Never/rarely
- Sometimes
- Often/always
- Don't know
- Refused
- Not applicable

### Penalties/Fines (P)

The next questions are about penalties for violations of laws regulating indoor tanning.

P1. If a tanning facility in [city/county] violates a law regulating this type of business, will the facility receive a citation?

- No **(skip the remaining items)**
- Yes
- Don't know
- Refused
- Not applicable

P2. In general, what is the penalty if a facility is cited for selling tanning sessions to underage youth?

P2a. A warning

- No
- Yes
- Don't know
- Refused
- Not applicable

P2b. A monetary fine

- No
- Yes
- Don't know
- Refused
- Not applicable

P2c. License suspension

- No
- Yes
- Don't know
- Refused
- Not applicable

P2d. Other **(probe)**

P3. If a facility is cited more than once for selling tanning sessions to underage youth, does [city/county] use graduated penalties, in which each repeat violation results in a larger penalty?

- No
- Yes
- Don't know
- Refused
- Not applicable

P4. In general, what are the penalties if a facility is cited for not obtaining parental permission for minors?

P4a. A warning

- No
- Yes
- Don't know
- Refused
- Not applicable

P4b. A monetary fine

- No
- Yes
- Don't know
- Refused
- Not applicable

P4c. License suspension

- No
- Yes
- Don't know
- Refused
- Not applicable

P4d. Other **(probe)**

P5. If a facility is cited more than once for not obtaining parental permission, does your city/county use graduated penalties, in which each repeat violation results in a larger penalty?

- No
- Yes
- Don't know
- Refused
- Not applicable

That concludes all my questions. I really appreciate all of your time! Do you have any questions or comments about this survey? Thanks again. Goodbye.
